# Optimization of Xiaoer Lingyin granule formulation using single-factor and response surface methodology

**DOI:** 10.1039/d6ra02969h

**Published:** 2026-07-14

**Authors:** Yanru Liu, Taoyang Cai, S. K. Kot-Cheung, Xinli Song, Feixiong Chen

**Affiliations:** a Guizhou University of Traditional Chinese Medicine Guiyang 550025 China 25034100672@stu.hebmu.edu.cn 392347047@qq.com; b Hebei Medical University Shijiazhuang 050017 China 25034100672@stu.hebmu.edu.cn; c Northeast Forestry University Harbin 150040 China cty@nefu.edu.cn OmniScience2025@126.com; d Faculty of Biochemistry and Molecular Medicine, University of Oulu Oulu 90014 Finland feixiong.chen@oulu.fi

## Abstract

This study aimed to develop and optimize Xiaoer Lingyin granules (XLGs), a pediatric traditional Chinese medicine formulation for acute pharyngitis, by integrating single-factor experiments, multi-index quality evaluation, and response surface methodology (RSM). Key formulation and process variables, including drug-to-excipient ratio, ethanol concentration, ethanol dosage, drying temperature, and drying time, were systematically evaluated to improve granule formability, flowability, dissolution performance, and palatability. The optimized formulation consisted of a drug-to-excipient ratio of 1 : 1, 65% (v/v) ethanol, 22.5% ethanol dosage, drying at 70 °C for 60 min, 0.2% sucralose, and 0.8% strawberry flavoring. Under these conditions, the granules showed high molding ratios, acceptable flowability, and dissolution ratios of ≥79.76%. The RSM model exhibited good predictive reliability, as confirmed by ANOVA, lack-of-fit analysis, *R*^2^-related statistics, and validation experiments. This study establishes a quantitative formulation optimization framework for pediatric TCM granules by integrating granulation quality, dissolution behavior, and sensory acceptability, providing a practical strategy for improving the standardization and patient compliance of traditional Chinese medicine preparations.

## Abstract 2

The study aimed to develop and optimize Xiaoer Lingyin granules (XLGs), a pediatric Chinese medicine formulation for acute pharyngitis, by integrating single-factor experiments, multi-index quality evaluation, and response surface methodology (RSM).

## Graphical abstract

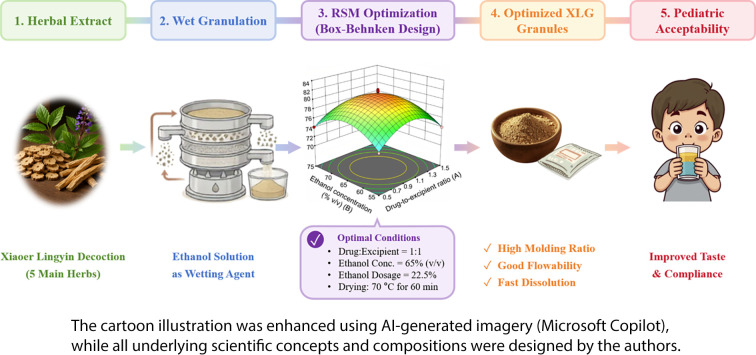

## 1 Introduction

Acute pharyngitis (AP) is a common upper respiratory tract infection in children and remains a frequent reason for clinical visits. Inappropriate or excessive use of antibiotics for pediatric respiratory infections may disturb the gut microbiota and contribute to antimicrobial resistance.^1–5^ Because children have immature immune systems and lower tolerance to adverse drug reactions, safer and more acceptable therapeutic options are needed. Traditional Chinese medicine (TCM) has been widely used as an adjunct or alternative approach for pediatric respiratory diseases because of its multi-component and symptom-oriented therapeutic characteristics.^6^

Xiaoer Lingyin Decoction is a clinically used formula from Guizhou University of Traditional Chinese Medicine and contains Smilax glabra, Commelina communis, and three additional herbs. It is used for pediatric AP because of its heat-clearing, detoxifying, and throat-soothing effects. Bioactive constituents such as astilbin and isoorientin have been reported to exhibit anti-inflammatory and immunomodulatory activities.^7,8^ The clinical application of this prescription in the Department of Pediatrics at the Second Affiliated Hospital of Guizhou University of Traditional Chinese Medicine has exceeded ten years, with a reported cure rate of 85% for pediatric acute pharyngitis, and no serious adverse reactions have been documented during this period. This historical clinical use provides preliminary empirical evidence for the safety and tolerability of the prescription in the pediatric population. However, the traditional decoction form is inconvenient for pediatric use because it requires repeated preparation, has poor portability, and often shows unpleasant taste. These limitations reduce medication adherence in children. Converting the decoction into granules can improve dose standardization, storage stability, portability, and administration convenience, but the formulation must simultaneously achieve acceptable granulation behavior, rapid dissolution, moisture resistance, and palatability.^9^

Previous studies have optimized TCM granules mainly by adjusting excipient type, excipient ratio, wetting agent concentration, or drying conditions. For example, single-factor experiments and response surface analysis have been used to improve molding rate, flowability, dissolution behavior, and hygroscopicity in different granule formulations.^10–12^ Nevertheless, most studies still focus on one or several physicochemical indicators, while the integrated optimization of pediatric granules remains insufficient. For pediatric TCM granules, formulation quality cannot be judged only by molding ratio or dissolution performance. Flowability, moisture resistance, taste masking, and sensory acceptability are also critical for storage stability, manufacturability, and patient compliance.^13–15^ Therefore, a quantitative multi-index evaluation strategy is needed to guide formulation design and reduce empirical trial-and-error in TCM granule development.

RSM is useful for evaluating interactions among formulation variables and identifying optimal process conditions. However, its application to pediatric TCM granules has often been limited to physicochemical optimization, with insufficient integration of granulation quality and patient-oriented sensory acceptability. This limits the translation of empirical TCM granulation experience into reproducible formulation design.

In this study, XLGs were developed and optimized by combining single-factor experiments, multi-index quality evaluation, sensory screening, and RSM. The main contribution of this work is the establishment of a quantitative formulation optimization framework for pediatric TCM granules. This framework integrates molding ratio, angle of repose, bulk density, dissolution ratio, and palatability-related sensory evaluation, allowing formulation variables to be assessed from both manufacturing and pediatric-use perspectives. By converting an empirical wet-granulation process into a statistically supported optimization strategy, this study provides a practical basis for improving the standardization, reproducibility, and clinical acceptability of XLGs.

## 2 Materials and methods

### 2.1 Materials

All medicinal materials were uniformly procured from Bohetang Chinese Herbal Pieces Co., Ltd. Specifically, *Belamcanda chinensis* (She Gan) and *Smilax glabra* (Tu Fu Ling) were produced in Hubei Province, *Mentha haplocalyx* (Bo He) in Anhui Province, *Commelina communis* (Ya Zhi Cao) in Yunnan Province, and *Gynura divaricata* (Guan Yin Cao) in Shaanxi Province.

Dextrin (Batch No. 20240910) and Microcrystalline Cellulose (Batch No. 3230514001) from Solarbio Science & Technology Co., Ltd. Aspartame (Batch No. A306C031) supplied by Solarbio Science & Technology Co., Ltd. Soluble Starch (Batch No. 20211006) sourced from Tianjin Zhiyuan Chemical Reagent Co., Ltd. Lactose (Batch No. C15217231) and Sucralose (Batch No. C15422524) provided by Shanghai Macklin Biochemical Technology Co., Ltd. Sodium Chloride (Batch No. 10019318) obtained from Sinopharm Chemical Reagent Co., Ltd. Stevioside (Batch No. 20231020) purchased from Qufu Shengren Pharmaceutical Co., Ltd. Flavoring agents such as Strawberry Flavor (Batch No. MH-J10003), Red Apple Flavor (Batch No. MH-J10011) and Sweet Orange Flavor (Batch No. MH-J10142) were produced by Shenzhen Longgang Piaoxiangyuan Trading Firm.

The excipients were selected according to their expected functional roles in wet granulation and pediatric administration. Dextrin, soluble starch, lactose, and microcrystalline cellulose were investigated as candidate diluents or carriers because they are commonly used to improve powder dispersibility, wet mass formability, granule cohesion, flowability, and dissolution behavior. In particular, dextrin was included as a potential carrier to reduce hygroscopicity and improve granule molding performance, whereas soluble starch and microcrystalline cellulose were evaluated for their swelling, water-binding, and structure-forming properties. Lactose was tested as a water-soluble diluent that may improve mouthfeel and dissolution. Ethanol–water mixtures were used as wetting agents to regulate liquid-bridge formation, wet mass plasticity, granule consolidation, and subsequent disintegration. Sodium chloride was used only to prepare a saturated salt environment for the hygroscopicity test and was not incorporated into the final formulation. Aspartame, sucralose, mannitol, and stevioside were screened as sweeteners to mask the bitterness and astringency of the herbal extract, while strawberry, red apple, and sweet orange flavors were evaluated to improve aroma and pediatric acceptability. All excipients used in this study, including dextrin, sucralose, and flavoring agents, are conventional pharmaceutical-grade materials widely employed in oral solid dosage forms. Dextrin is commonly used as a diluent and carrier in granule formulations; sucralose is a non-caloric sweetener approved for food and pharmaceutical applications (GB 2760, China; FDA GRAS); and flavoring agents are routinely incorporated to improve palatability and patient compliance in pediatric formulations. The use of these excipients is consistent with established pharmaceutical guidelines and does not introduce novel chemical entities or modify the intrinsic structure of the herbal active constituents. Therefore, the excipient screening was designed to balance manufacturability, physical stability, dissolution performance, and palatability rather than to select excipients empirically.

Although the present study does not include dedicated toxicological or pediatric clinical trials, the safety profile of the formulation is supported by multiple levels of evidence. First, Xiaoer Lingyin Decoction has been clinically applied in pediatric respiratory conditions for over ten years in a hospital setting, with no reported serious adverse reactions, providing preliminary real-world safety evidence. Second, all excipients used in this study are conventional pharmaceutical-grade materials widely used in oral solid dosage forms and are recognized as safe under relevant regulatory frameworks, including GB 2760 food additive standards and FDA GRAS classification for applicable substances. Importantly, the incorporation of these excipients does not alter the intrinsic chemical composition of the herbal constituents, ensuring that the formulation remains chemically consistent with the original prescription.

Therefore, the present work focuses on process optimization rather than new drug development, and the observed improvements in granule properties are attributed to physical formulation engineering rather than chemical modification of active components. However, systematic preclinical toxicology studies and controlled clinical evaluations remain necessary to fully establish the safety and therapeutic profile of the optimized pediatric granules.

### 2.2 Preparation of XLGs

**Fig. 1:**
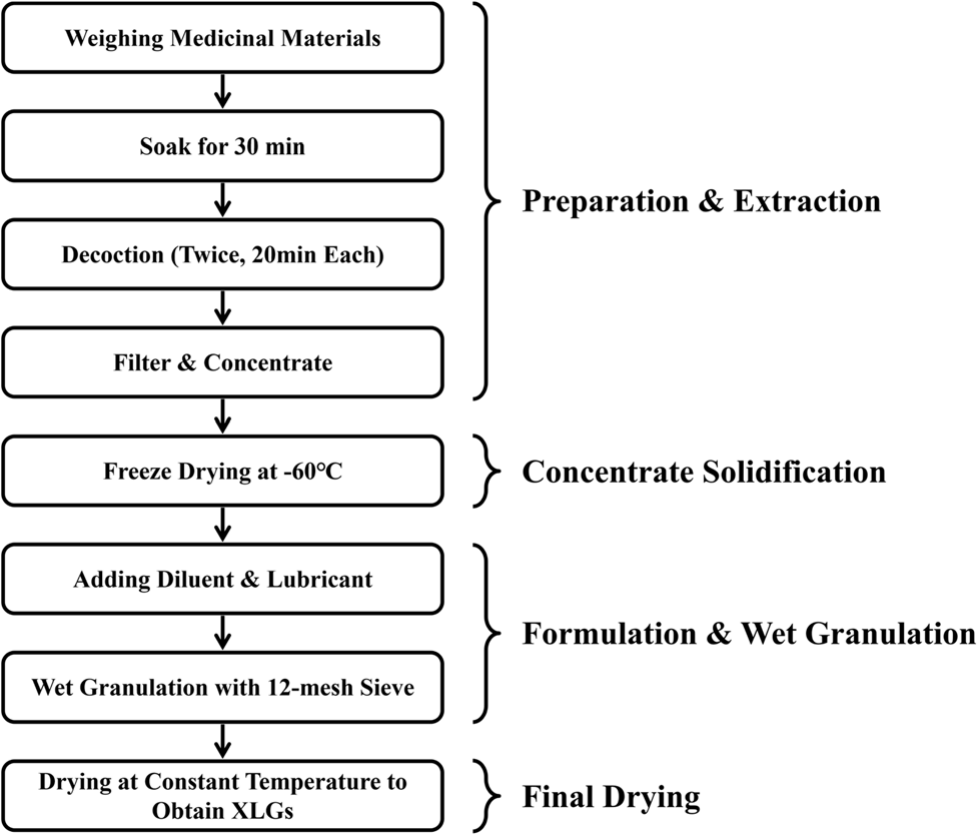
Flow chart of XLGs preparation.

Weighing the medicinal materials according to the prescription, soaking for 30 minutes, decocting for 20 minutes, decocting twice, filtering, concentrating, and freeze-drying at −60 °C to obtain freeze-dried powder for later use. The granules were prepared by wet granulation process.^16^ The auxiliary materials and the medicinal powder are evenly mixed with the main medicine in a mortar, and a proper amount of wetting agent is added to prepare a soft material. When the color of the soft material becomes dark, the soft material is “kneaded into a ball and dispersed on contact”, and the soft material is wet granulated by a 12-mesh granulating screen and dried in an air-forced drying oven under the designed drying conditions, with drying temperature and drying time optimized in the single-factor experiments, thus obtaining the XLGs.^17^ The preparation process is shown in Fig. 1. The endpoint of wet massing was controlled by the physical state of the soft material rather than by a fixed mixing time; the qualified wet mass was defined as being able to be kneaded into a ball by hand and readily dispersed upon gentle pressure.

#### 2.2.1 Preparation of dry extract powder by different drying methods

For comparison of drying methods, the concentrated Xiaoer Lingyin decoction was divided into three portions and subjected to freeze-drying (−60 °C, 31 Pa), vacuum drying (70 °C, −0.08 MPa), and atmospheric drying (105 °C), respectively. The resulting dry extract powders were ground, passed through an 80-mesh sieve, and subjected to physical fingerprint analysis including bulk density (*D*_a_), tapped density (*D*_c_), Hausner ratio (*I*_H_), Carr's index (*I*_C_), angle of repose (*α*), moisture content (MC), hygroscopicity (*H*), critical relative humidity (CRH), and interparticle porosity (*I*_e_) according to established methods.

#### 2.2.2 Chemical characterization and fingerprint analysis

To evaluate the chemical quality consistency of XLGs prepared under different processing conditions, four representative marker compounds, namely neoastilbin, astilbin, irigenin, and irisflorentin, were quantitatively determined using UPLC analysis. In addition, chromatographic fingerprint profiles were established to assess overall chemical similarity among samples.

##### 2.2.2.1 UPLC conditions

Chromatographic analysis was performed using an Agilent 1290 UPLC system equipped with a Waters ACQUITY HPLC® C18 column (100 mm × 2.1 mm, 1.7 µm). The mobile phase consisted of 0.1% formic acid in water (A) and acetonitrile (B), applied under a gradient elution program as follows: 0–10 min, 5–10% (B); 11–15 min, 10–15% (B); 16–20 min, 15–18% (B); 21–26 min, 18–20% (B); 27–28 min, 20–21% (B); 29–35 min, 21–22% (B); 36–40 min, 22–24% (B); 41–43 min, 24–26% (B); 44–45 min, 26–28% (B); 46–48 min, 28–30% (B); 49–50 min, 30–35% (B); 51–55 min, 35–65% (B); and 56–60 min, 65–95% (B). The column temperature was maintained at 30 °C, the flow rate was set at 0.15 mL min^−1^, the injection volume was 4 µL, and detection was carried out at 286 nm.

##### 2.2.2.2 Preparation of standard solutions

Reference standards of neoastilbin, astilbin, irigenin, and irisflorentin (purity ≥ 98%) were accurately weighed and dissolved in 50% methanol to prepare individual stock solutions. Working mixed standard solutions were obtained by appropriate dilution and combination of each stock solution using 50% methanol.

##### 2.2.2.3 Preparation of sample solutions

For granule samples, approximately 1.0 g of XLGs was accurately weighed and transferred into a 10 mL volumetric flask. The sample was dissolved in 50% methanol, sonicated for 30 min, cooled to room temperature, and filtered through a 0.22 µm membrane prior to analysis. For decoction reference samples, 2.0 mL of concentrated decoction was diluted to 10 mL with 50% methanol and processed under the same conditions.

##### 2.2.2.4 Method validation

The analytical method was validated in accordance with ICH guidelines, including assessments of linearity, precision, repeatability, stability (24 h), and recovery. All four marker compounds exhibited excellent linearity within their respective concentration ranges, with correlation coefficients (*R*^2^) greater than 0.9995.

##### 2.2.2.5 Fingerprint analysis

UPLC chromatographic data from nine batches of XLGs (three batches per drying method) were analyzed using the “Similarity Evaluation System for Chromatographic Fingerprint of Traditional Chinese Medicine (2012 Edition)”. The freeze-dried sample (S1) was selected as the reference chromatogram. Peak alignment was performed using multi-point calibration with a time window of 0.1 min. The similarity between each sample and the reference fingerprint was calculated to evaluate overall chemical consistency among different preparation batches.

### 2.3 Evaluation index and standard of granule prescription

The granule quality evaluation methods were established based on commonly used quality attributes of pharmaceutical granules, including hygroscopicity, molding ratio, powder flowability, bulk density, dissolution behavior, and comprehensive score.^18–22^

#### 2.3.1 Hygroscopicity

A supersaturated NaCl solution was placed in a desiccator for 48 hours at room temperature to maintain 75% relative humidity.^23,24^ Pre-dried weighing bottles were loaded with 0.5 g of granules (spread uniformly, thickness ≤5 mm), dried to constant weight at 60 °C, and then placed uncovered in the desiccator. Mass changes were recorded at 0, 6, 12, 24, 36, 48, and 60 hours. All hygroscopicity measurements were performed in triplicate, and the results were expressed as mean ± standard deviation. Statistical analysis was conducted to evaluate the reproducibility and variability of the measurements. Hygroscopicity was calculated as:
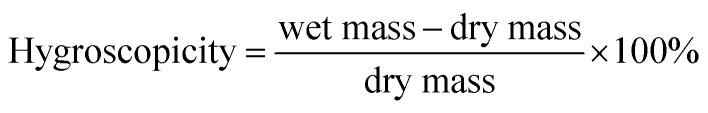


#### 2.3.2 Molding ratio

The molding ratio of granules was evaluated using a dual-sieve method. In this method, a No. 1 sieve served as the upper sieve and a No. 5 sieve as the lower sieve. A defined quantity of granules was placed on the upper sieve, covered, and shaken uniformly for 3 minutes. After shaking, the masses of oversized granules retained on the No. 1 sieve and undersized powder passing through the No. 5 sieve were separately weighed. The molding ratio was calculated using the formula:
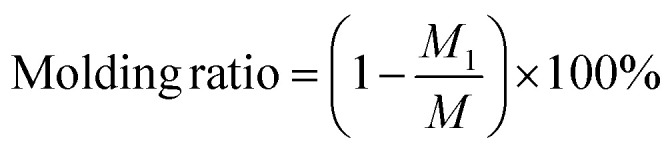
where *M* is the total mass of granules, and *M*_1_ is the combined mass of oversized granules and undersized powder.

#### 2.3.3 Angle of repose

The angle of repose was determined *via* a fixed funnel method. Three funnels were vertically aligned and fixed on an iron stand. The distance between the funnel outlet and the coordinate paper was adjusted to 5.0 cm using a ruler. Granules were slowly poured along the inner wall of the top funnel until the tip of the formed cone touched the funnel outlet. The height (*H*) and radius (*r*) of the granule cone were measured to calculate the angle of repose (*α*):
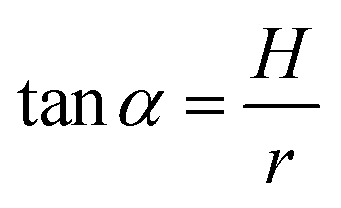
when 0° ≤ *α* ≤ 30°, score 100 points; when 30° < *α* ≤ 40°, score 80 points; when *α* > 40°, score 60 points.

#### 2.3.4 Bulk density

A precisely weighed powder sample (sieved through a 1.0 mm mesh to disperse agglomerates formed during storage) was gently poured into a graduated glass cylinder without compaction. The apparent volume (*V*_0_) was recorded at the nearest scale mark. Bulk density was calculated as:
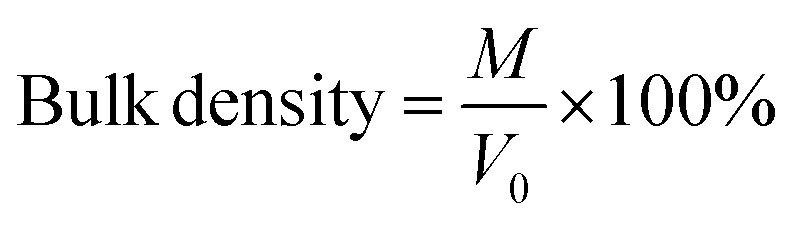
where *M* is the mass of the powder (g), and *V*_0_ is the apparent volume (mL). Triplicate measurements were averaged for final results.

#### 2.3.5 Dissolution ratio

Dried granules (0.5 g) were placed in a test tube, mixed with 5 mL hot water *via* vortexing for 5 minutes, and centrifuged at 3500 rpm for 10 minutes. The supernatant was removed, and the residue was dried to constant weight at 60 °C. The dissolution ratio was calculated as:
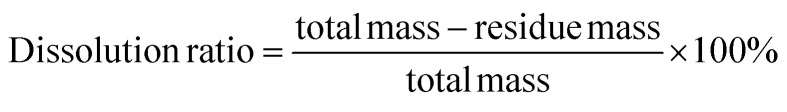


#### 2.3.6 Comprehensive score

The comprehensive score was used to integrate multiple quality attributes into a single response value for formulation comparison, as reported in previous multi-index evaluation studies of TCM preparations and formulation optimization.^25^ The score was derived from four parameters: molding ratio (a), angle of repose score (b), bulk density (c), and dissolution ratio (d). Equal weight was assigned to the four indicators to avoid overemphasizing a single quality attribute and to provide a simple comparative evaluation during preliminary formulation screening. The formula was as follows:
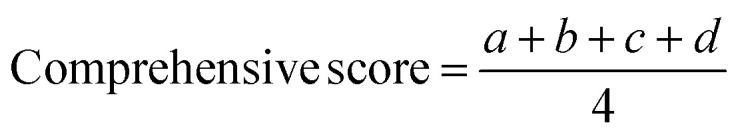


### 2.4 Single-factor method screening

Single-factor experiments were performed to preliminarily determine suitable ranges of formulation and process variables before RSM optimization. This step is commonly used in formulation studies to identify influential factors, narrow the experimental range, and reduce unnecessary experimental runs in subsequent statistical optimization.^26–28^ In each experiment, only one variable was changed, while the other preparation parameters were kept constant. Each formulation group was prepared and measured in triplicate. Molding ratio, angle of repose score, bulk density, dissolution ratio, and comprehensive score were used as evaluation indicators. The factor level with the highest comprehensive score and acceptable granule appearance was selected for subsequent optimization.

#### 2.4.1 Diluent selection

Microcrystalline cellulose, soluble starch, lactose, and dextrin were selected as candidate diluents or carriers based on their different physicochemical functions in wet granulation.^29–31^ Microcrystalline cellulose was evaluated for its water-binding capacity and ability to improve granule structure and flowability. Soluble starch was tested because of its swelling and disintegration-promoting properties. Lactose was included as a water-soluble diluent with potential benefits for dissolution and mouthfeel. Dextrin was evaluated as a carrier with potential advantages in reducing hygroscopicity, improving powder dispersibility, and enhancing wet mass formability. Xiaoer Lingyin powder and each excipient were mixed at a mass ratio of 1 : 1 and granulated under identical wet granulation conditions. Hygroscopicity at 25 °C and 75% relative humidity, together with granule appearance and formability, was used as the primary screening basis. Each group was prepared and tested in triplicate.

#### 2.4.2 Diluent ratio

To evaluate the effect of drug-to-excipient ratio on granulation performance, five ratios were investigated: 0.5 : 1, 1 : 1, 1.5 : 1, 2 : 1, and 2.5 : 1. The ethanol concentration, ethanol dosage, drying temperature, and drying time were kept constant during this screening step. Granules were prepared using the same wet granulation procedure, and the optimal ratio was determined based on the comprehensive score calculated from molding ratio, angle of repose, bulk density, and dissolution ratio. Each group was tested in triplicate.

#### 2.4.3 Wetting agent concentration

Ethanol–water mixtures were used as wetting agents to regulate the viscosity, plasticity, and liquid-bridge formation of the wet mass during granulation.^17,32,33^ Five ethanol concentrations, namely 55%, 65%, 75%, 85%, and 95% (v/v), were investigated. During this experiment, the drug-to-excipient ratio, wetting agent dosage, drying temperature, and drying time were kept constant. Each wet mass was prepared using the same mixing and sieving procedure, and the resulting granules were evaluated using molding ratio, angle of repose score, bulk density, dissolution ratio, and comprehensive score. The ethanol concentration that produced the highest comprehensive score and acceptable wet mass formability was selected for subsequent experiments.

#### 2.4.4 Wetting agent dosage

After determining the optimal ethanol concentration, the effect of wetting agent dosage on granule quality was further investigated. Five wetting agent dosages, ranging from 7.5% to 27.5% with an interval of 5%, were tested. The drug-to-excipient ratio, ethanol concentration, drying temperature, and drying time were kept constant. The wetting agent was added gradually during mixing to avoid local overwetting and to obtain a uniform wet mass. The prepared wet granules were dried and sieved under identical conditions. Molding ratio, angle of repose score, bulk density, dissolution ratio, and comprehensive score were used to determine the appropriate wetting agent dosage.^19^

#### 2.4.5 Drying temperature

Drying temperature was optimized because it can affect residual moisture, granule strength, porosity, and dissolution behavior.^34,35^ Wet granules were placed in an air-forced drying oven and dried at 60 °C, 70 °C, or 80 °C for 60 min. Other formulation and process parameters were kept constant. After drying, the granules were cooled to room temperature in a desiccator and then sieved. Granule appearance, molding ratio, angle of repose score, bulk density, dissolution ratio, and comprehensive score were evaluated. The drying temperature that provided the best balance between granule formability and dissolution performance was selected for the next step.

#### 2.4.6 Drying time

Based on the selected drying temperature, the effect of drying time was further evaluated. Wet granules were dried for 60, 75, or 90 min in an air-forced drying oven while keeping the formulation composition and other process parameters unchanged. After drying, the granules were cooled to room temperature and sieved before quality evaluation. Drying time was optimized according to granule appearance, molding ratio, angle of repose score, bulk density, dissolution ratio, and comprehensive score. This evaluation was performed because insufficient drying may cause adhesion and poor flowability, whereas excessive drying may alter granule porosity and reduce dissolution performance.^35^

#### 2.4.7 Screening of sweeteners and flavoring agents

Mannitol, sucralose, stevioside, and aspartame were screened as sweeteners because the herbal extract had a characteristic bitter and astringent taste that may reduce pediatric compliance. These sweeteners were selected to compare differences in sweetness intensity, aftertaste, taste-masking ability, and dosage requirement.^36^ Strawberry, red apple, and sweet orange flavors were further evaluated as aroma-modifying agents to improve the overall sensory acceptability of XLGs.

A single-blind randomized sensory evaluation was conducted with 10 healthy volunteers who had fasted for 2 h before testing. Each sample was prepared under the same granulation conditions except for the type and dosage of the sweetener or flavoring agent. The volunteers evaluated smell, sweetness, bitterness, and astringency using a 0–3 scoring scale, where a higher score indicated better sensory acceptability. The additive and dosage that achieved the highest average sensory score with the lowest practical amount were selected for the final formulation.

#### 2.4.8 Caregiver-reported acceptability survey

To further evaluate pediatric-use suitability from a real-use perspective, an anonymous questionnaire-based acceptability survey was conducted among caregivers of children who had previously used Xiaoer Lingyin Decoction for pediatric acute pharyngitis. The survey aimed to assess caregiver-reported administration difficulties associated with the traditional decoction and the expected acceptability of converting the decoction into taste-masked granules.

Eligible respondents were caregivers of children with prior experience using Xiaoer Lingyin Decoction. The survey was reviewed and approved by the Ethics Committee of Guizhou University of Traditional Chinese Medicine (Approval No. 2024064). Written or electronic informed consent was obtained from all respondents before questionnaire completion. The survey was anonymous and collected no personally identifiable information, including medical record numbers, telephone numbers, or home addresses. Only non-identifiable information was collected, including the child's age range, frequency of previous decoction use, caregiver relationship to the child, and caregiver-reported experience during administration.

The questionnaire consisted of three parts. The first part evaluated real-use problems of the traditional decoction, including bitterness/astringency, large administration volume, preparation inconvenience, storage/portability limitations, and child refusal or delayed intake. The second part assessed caregiver preference for granule conversion, including perceived convenience of granules, willingness to choose a granule formulation, and perceived improvement in pediatric compliance after taste masking. The third part evaluated flavor preference and acceptability of sweetener/flavoring addition. Items were scored using a five-point Likert scale, where 1 indicated “strongly disagree” and 5 indicated “strongly agree”.

For each item, the mean score and the percentage of respondents selecting “agree” or “strongly agree” (scores 4 and 5) were calculated. Data are presented as *n* (%) and mean ± SD.

### 2.5 Response surface methodology for prescription optimization

#### 2.5.1 Experimental design

Based on the single-factor experimental results, three critical variables were selected for RSM optimization: drug-to-excipient ratio, ethanol concentration, and ethanol dosage. RSM with a Box–Behnken design was used because it can evaluate linear, quadratic, and interaction effects among formulation variables with fewer experimental runs than full-factorial designs. A Box–Behnken design with 17 experimental runs, including five center-point replicates, was employed to investigate the effects of these three variables on granule quality. Each formulation was prepared and evaluated in triplicate to improve the reliability and repeatability of the results. The comprehensive score, calculated from molding ratio, angle of repose score, bulk density, and dissolution ratio, was used as the response value.

#### 2.5.2 Model construction

The experimental data were analyzed using Design-Expert 13.0 software (Stat-Ease Inc., Minneapolis, USA). A second-order polynomial model was fitted to describe the relationship between the independent variables and the comprehensive score. The statistical significance of the overall model, linear terms, interaction terms, and quadratic terms was evaluated by analysis of variance (ANOVA), as commonly applied in RSM-based formulation optimization. Model adequacy was assessed using the lack-of-fit test, coefficient of determination (*R*^2^), adjusted *R*^2^, predicted *R*^2^, coefficient of variation (C.V.), adequate precision, and residual diagnostic plots. Statistical significance was defined as *p* < 0.05.

#### 2.5.3 Validation protocol

The optimal formulation conditions predicted by the RSM model were validated through three independent experimental batches. For each batch, XLGs were prepared according to the adjusted optimal conditions and evaluated using the same quality indicators described above. The experimental comprehensive scores were compared with the predicted value generated by the fitted model. The relative error was calculated to assess the predictive accuracy and practical reliability of the model. A small relative error between predicted and experimental values was considered evidence that the optimized formulation conditions were reproducible and suitable for subsequent preparation.^37^

### 2.6 Preliminary stability evaluation

To preliminarily evaluate the stability of the optimized XLGs, three independent batches of optimized freeze-dried granules were stored under ambient conditions (25 ± 2 °C and 60 ± 5% relative humidity). Samples were collected at 0, 7, and 14 days for UPLC fingerprint analysis, marker compound quantification, and moisture content determination. For moisture content measurement, approximately 0.5 g of granules was accurately weighed and dried at 60 °C to constant weight. Moisture content was calculated as the percentage of weight loss relative to the initial sample weight. All measurements were performed in triplicate, and the results are expressed as mean ± standard deviation.

## 3 Results and discussion

### 3.1 Screening results of diluents

**Fig. 2:**
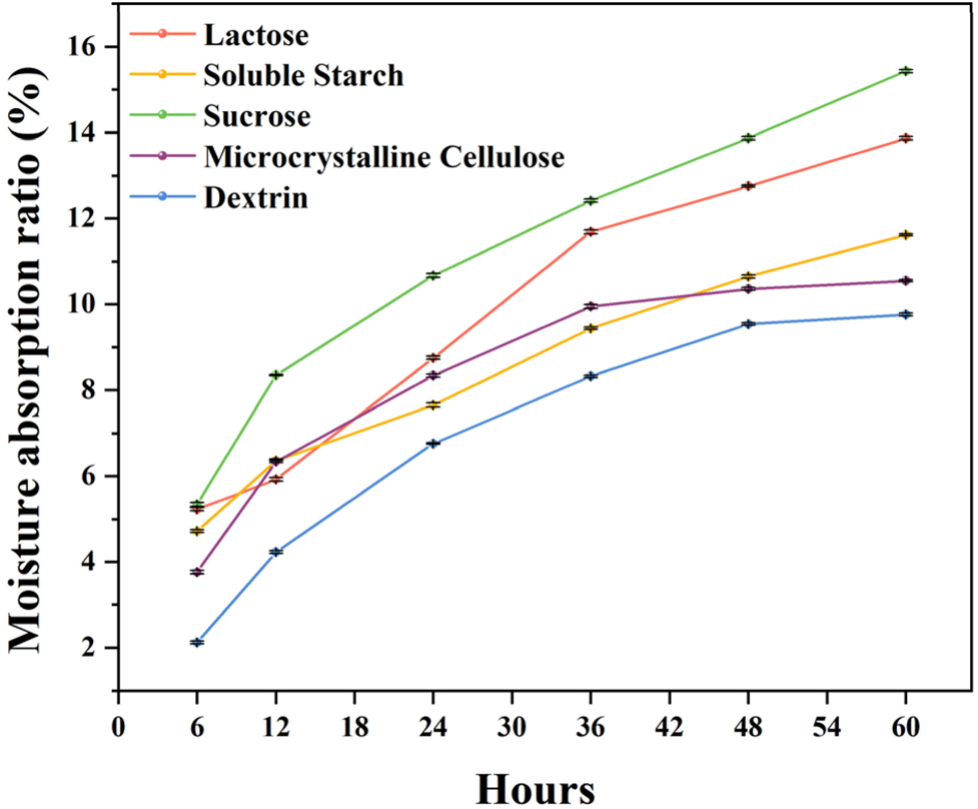
Moisture absorption ratio (%) of diluents over 6–60 hours, with mean ± standard deviation, *n* = 3.

An appropriate amount of Xiaoer Lingyin decoction powder was uniformly mixed with five diluents (soluble starch, dextrin, sucrose, lactose, and microcrystalline cellulose) at a drug-to-excipient ratio of 1 : 1. The wet mass was prepared using 60% ethanol as the wetting agent, followed by granulation through a 12-mesh sieve, drying at 60 °C, and sieving to obtain XLGs. When lactose was used as the diluent, the resulting granules exhibited excessive viscosity, poor cohesiveness, and moderate formability. Granules prepared with sucrose, soluble starch, or microcrystalline cellulose failed to meet appearance standards. In contrast, dextrin as the diluent facilitated uniform granule formation with optimal shape and formability. As shown in Fig. 2, the hygroscopicity of the mixture containing Xiaoer Lingyin powder and dextrin was the lowest (9.76% at 60 h). Therefore, dextrin was selected as the optimal diluent for XLGs. The superior performance of dextrin may be attributed to its moderate molecular weight and partial amorphous structure, which enhance inter-particle hydrogen bonding and improve granule cohesion, while its relatively low hygroscopicity reduces moisture uptake and prevents agglomeration. These characteristics explain the observed balance between flowability and stability in XLGs. Our findings are consistent with previous reports that highlighted dextrin as an effective carrier for stabilizing herbal granules and improving molding performance.^38,39^ However, unlike studies where dextrin was mainly employed to control dissolution or hygroscopicity, our work further demonstrates its synergistic role in optimizing both physical formability and sensory attributes, suggesting broader applicability in TCM granule formulations.

#### 3.1.1 Physical fingerprint analysis of dry extracts prepared by different drying methods

To further support the observed differences in powder behavior among drying methods, the physicochemical properties of herbal extract powders were further analyzed. Freeze-drying typically generates highly porous, low-density powder structures characterized by reduced interparticle cohesion and improved flowability, which are beneficial for subsequent granulation processes. Consistent with common findings, the freeze-dried Xiaoer Lingyin extract exhibited favorable moisture resistance and powder stability, as reflected by its low hygroscopicity and improved flow-related performance.

**Fig. 3:**
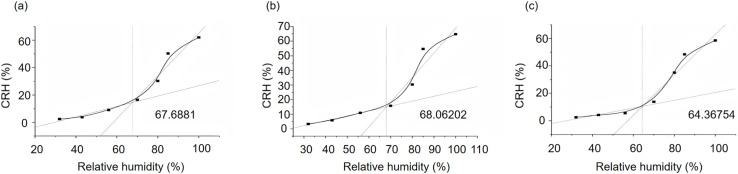
Critical relative humidity (CRH) curves of Xiaoer Lingyin dry extract powders prepared by different drying methods. (a) Freeze-dried sample (CRH = 67.69%); (b) vacuum-dried sample (CRH = 68.06%); (c) atmospheric-dried sample (CRH = 64.37%). The CRH was determined as the intersection point of the moisture sorption curve (solid line with data points) and the diagonal reference line (dashed line).

**Fig. 4:**
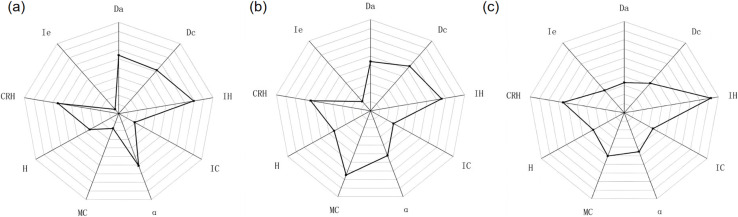
Physical fingerprint radar charts of Xiaoer Lingyin dry extract powders prepared by different drying methods. (a) Freeze-dried sample; (b) vacuum-dried sample; (c) atmospheric-dried sample. The nine axes represent standardized scores for bulk density (*D*_a_), tapped density (*D*_c_), Hausner ratio (*I*_H_), Carr's index (*I*_C_), angle of repose (*α*), moisture content (MC), hygroscopicity (*H*), critical relative humidity (CRH), and interparticle porosity (*I*_e_).

The integrated powder behavior profile (including hygroscopicity trend, CRH response, and comprehensive physical fingerprint scoring) is summarized in Table 1, while the moisture sorption behavior and stability profiles are shown in Fig. 3. The overall physical fingerprint (Fig. 4) results showed that freeze-dried and vacuum-dried extracts both exhibited acceptable powder properties for subsequent wet granulation. Although the vacuum-dried extract showed a slightly higher total physical fingerprint score, the freeze-dried extract maintained favorable porosity-related characteristics and moisture-related behavior. More importantly, the subsequent chemical characterization demonstrated that freeze-drying better preserved the representative marker compounds of the original decoction. Therefore, freeze-drying was selected as the preferred drying method by considering both physical suitability and chemical integrity.

**Table 1:** Standardized scores of physical fingerprint parameters for Xiaoer Lingyin dry extract powders prepared by different drying methods^a^

Indicator	Freeze drying	Vacuum drying	Atmospheric drying
*D*_a_	4.30	6.40	7.40
*D*_c_	5.22	7.38	7.20
*I*_H_	10.20	8.55	8.95
*I*_C_	4.47	3.78	2.92
*α*	5.53	6.22	7.08
MC	6.03	8.48	2.73
*H*	4.74	5.39	4.50
CRH	7.51	7.36	7.46
*I*_e_	4.20	2.33	1.60
Total score	52.20	55.89	49.84

a The values are dimensionless standardized scores used for physical fingerprint comparison. The total score reflects the overall powder physical profile and was used as a supplementary indicator rather than the sole criterion for selecting the drying method. The final selection of freeze-drying was based on combined consideration of physical suitability and chemical marker retention.

#### 3.1.2 Chemical composition and fingerprint consistency of dry extracts from different drying methods

To ensure that the optimized granulation process preserves the bioactive constituents of the original decoction, the contents of four representative marker compounds (neoastilbin, astilbin, irigenin, and irisflorentin) were quantified, and UPLC fingerprint chromatograms were established for granules prepared by freeze-drying, vacuum drying, and atmospheric drying.

As shown in Table 2, the freeze-dried granules exhibited the closest agreement with the decoction equivalent for all four marker compounds, with no statistically significant differences (*P* ≥ 0.05). The vacuum-dried granules showed only a slight decrease in irigenin content, whereas the atmospheric-dried granules exhibited significant reductions in neoastilbin, astilbin, and irisflorentin. Among these compounds, irisflorentin showed the most pronounced loss, decreasing by approximately 48.8% relative to the decoction equivalent. These results indicate that freeze-drying better preserved the representative marker compounds of the herbal extract, whereas high-temperature atmospheric drying caused substantial degradation of several heat-sensitive constituents.

**Table 2:** Content of four marker compounds in Xiaoer Lingyin decoction equivalent and granules prepared by different drying methods (mg g^−1^, *x̄* ± *s*, *n* = 3)

Sample	Neoastilbin (%)	Astilbin (%)	Irigenin (%)	Irisflorentin (%)
Decoction	0.4175 ± 0.0071	1.1864 ± 0.0065	0.1637 ± 0.0121	0.0244 ± 0.0036
Freeze-dried granules	0.4158 ± 0.0040	1.1806 ± 0.0052	0.1630 ± 0.0037	0.0241 ± 0.0046
Vacuum-dried granules	0.4155 ± 0.0156	1.1773 ± 0.0028	0.1522 ± 0.0044^a^	0.0231 ± 0.0028
Atmospheric-dried granules	0.3502 ± 0.0039^a^	1.1482 ± 0.0035^a^	0.1505 ± 0.0057^a^	0.0125 ± 0.0029^a^

a *P* < 0.05 *vs.* decoction equivalent. The decoction group was normalized to the equivalent dry extract basis for comparison with granule samples.

**Fig. 5:**
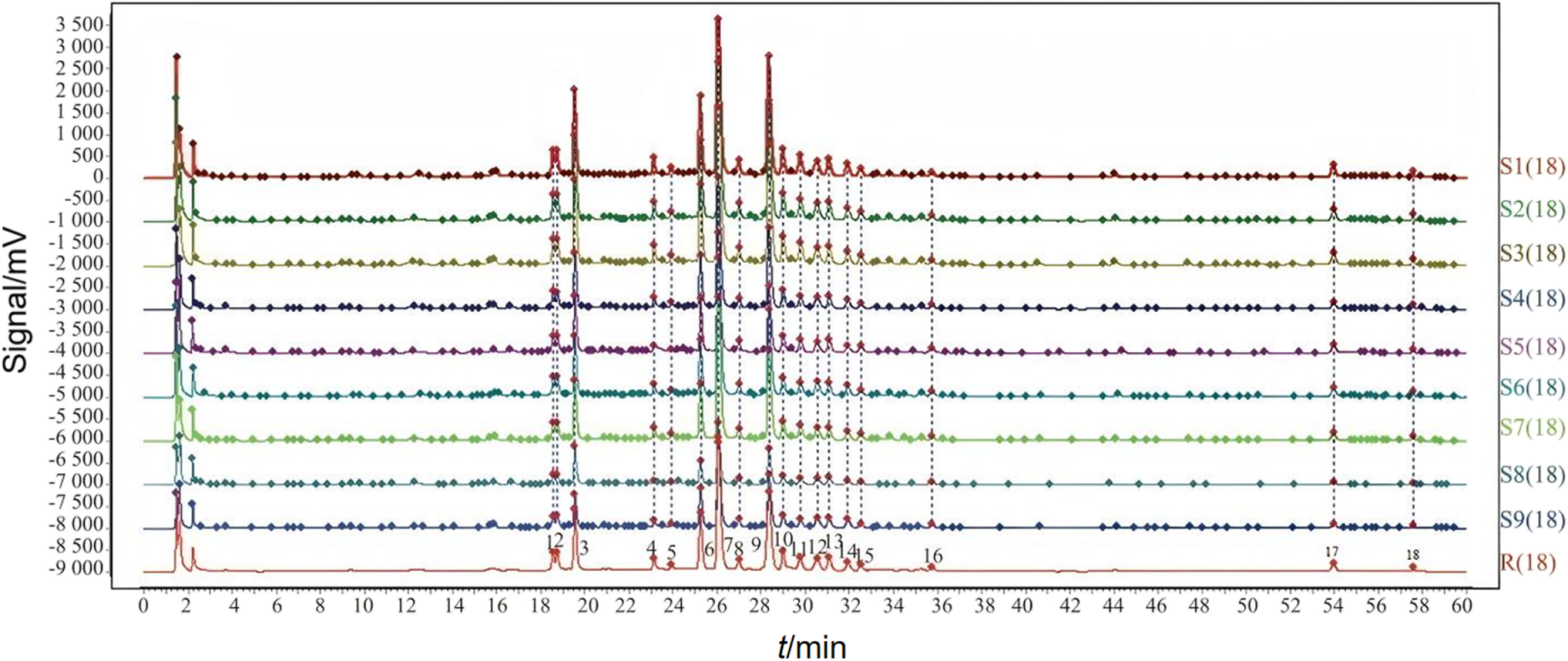
Superimposed UPLC fingerprint chromatograms of nine batches of Xiaoer Lingyin granule samples. (S1–S3): freeze-dried granule samples; (S4–S6): vacuum-dried granule samples; (S7–S9): atmospheric-dried granule samples. *R*(18) represents the reference fingerprint generated from 18 common peaks. Peaks 6, 7, 8, 9, 12, 13, 17, and 18 were assigned to neoastilbin, astilbin, tectoridin, naringin, engeletin, iridin, irigenin, and irisflorentin, respectively.

Fingerprint analysis further supported these findings (Fig. 5). The UPLC fingerprint chromatograms of nine batches of granules (three batches for each drying method) revealed 18 common peaks. Among them, eight representative peaks were assigned to known constituents, including neoastilbin, astilbin, tectoridin, naringin, engeletin, iridin, irigenin, and irisflorentin. Similarity evaluation indicated that all batches showed high similarity to the reference fingerprint (0.989–0.999), demonstrating good overall chemical consistency across drying methods. However, peak area comparisons revealed that atmospheric-dried samples had noticeably reduced peak intensities for heat-sensitive components, consistent with the quantitative results.

These chemical characterization data provide critical support for the selection of freeze-dried extract as the intermediate material for XLG preparation, ensuring both physical suitability and chemical integrity of the final formulation.

### 3.2 Optimization of drug-to-excipient ratio

To optimize the drug-to-excipient ratio, five gradient groups (0.5 : 1, 1 : 1, 1.5 : 1, 2 : 1, and 2.5 : 1) were designed for wet granulation. Key quality attributes, including molding ratio, angle of repose score, bulk density, and dissolution ratio, were quantitatively evaluated, and a comprehensive scoring system was used to compare the overall formulation performance.

As shown in Table 3, the 1 : 1 drug-to-excipient ratio provided the most balanced granulation performance among the tested groups. This formulation achieved a high molding ratio of 94.36%, an angle of repose score of 100, a bulk density of 49.01 g/100 mL, a dissolution ratio of 81.03%, and the highest comprehensive score of 81.10. In comparison, the comprehensive scores decreased when the drug proportion was further increased, with values of 79.35, 75.69, and 73.28 for the 1.5 : 1, 2 : 1, and 2.5 : 1 groups, respectively. The 0.5 : 1 group also showed a lower comprehensive score of 76.94, mainly due to its markedly reduced molding ratio of 48.72%.

**Table 3:** Effects of diluent-to-drug ratios on granulation performance

Drug-to-excipient ratio	Molding ratio (%)	Angle of repose score	Bulk density (g/100 mL)	Dissolution ratio (%)	Comprehensive score
0.5 : 1	48.72	100	44.09	76.13	76.94
1 : 1	94.36	100	49.01	81.03	81.10
1.5 : 1	91.90	100	47.28	78.22	79.35
2 : 1	83.15	100	43.99	75.61	75.69
2.5 : 1	77.08	100	46.00	70.02	73.28

These quantitative results support the selection of the 1 : 1 drug-to-excipient ratio. At this ratio, the formulation maintained sufficient excipient content to improve wet mass formability and granule formation, while also preserving adequate drug loading and dissolution performance. From a physicochemical perspective, the 1 : 1 ratio may provide sufficient excipient surface area to disperse drug particles and reduce interparticle friction, thereby improving flowability and granule uniformity.^40–42^ At the same time, excessive drug loading at higher ratios may reduce granule formability and dissolution performance, whereas excessive excipient content at the lower ratio may dilute the formulation and reduce molding efficiency. Therefore, the 1 : 1 drug-to-excipient ratio was selected as the optimal ratio based on multi-index quantitative evaluation rather than a single parameter.

### 3.3 Optimization of wetting agent concentration

Based on single-factor screening results, ethanol was selected as the wetting agent, and its concentration was investigated for its impact on granulation. With a fixed drug-to-dextrin ratio (1 : 1), wet granulation was performed using gradient ethanol concentrations (55–95%). A comprehensive evaluation system was established, incorporating granulation yield, angle of repose, bulk density, and dissolution rate as key metrics.

As shown in Table 4, the 65% ethanol group achieved the highest comprehensive score among the tested ethanol concentrations. At this concentration, molding ratio (95.15%) and dissolution ratio (80.62%) remained at high levels, while bulk density (50.16 g/100 mL) surpassed that of the 55% ethanol group, suggesting that moderate wetting enhances particle density. However, increasing ethanol concentration to 85% led to a sharp decline in dissolution rate (62.56%, a 22.4% reduction compared to the 65% group), despite further increases in bulk density (60.80 g/100 mL). This indicates that excessive ethanol may reduce material porosity, thereby inhibiting granule disintegration. The ranking of comprehensive scores confirmed that the 65% ethanol group achieved an optimal balance among granule formability, flowability, and rapid solubility. Mechanistically, ethanol at 65% (v/v) achieves an ideal polarity balance, it is strong enough to partially solubilize herbal constituents and generate adhesive liquid bridges for cohesive granule formation, while retaining sufficient water content to promote porosity and rapid disintegration. In contrast, lower ethanol concentrations fail to provide adequate binding strength, whereas higher concentrations reduce porosity and slow down dissolution.

**Table 4:** Effects of ethanol concentration on granulation performance

Ethanol concentration (%)	Molding ratio (%)	Angle of repose score	Bulk density (g/100 mL)	Dissolution ratio (%)	Comprehensive score
55	98.35	100	43.51	70.31	78.04
65	95.15	100	50.16	79.01	81.08
75	96.13	100	46.36	62.52	76.25
85	96.13	100	60.80	62.56	79.87
95	92.89	100	44.22	70.02	76.78

### 3.4 Optimization of wetting agent dosage

A single-factor experiment was conducted to investigate the effects of ethanol dosage on granule quality. As shown in Table 5, the comprehensive score reached 78.75 at an ethanol dosage of 22.5%, outperforming lower dosage groups. Compared to the 12.5% dosage group, the 22.5% group exhibited a 33.8% increase in granulation yield (97.99%) and a 14.3% improvement in bulk density (0.49 g/100 mL), while maintaining stable dissolution performance (67.58%). Although the 27.5% high-dosage group achieved comparable granulation yield (96.22%) to the 22.5% group, its bulk density decreased by 6.5%, indicating that excessive ethanol may lead to loose granule structure due to reduced cohesion.^43,44^ The ranking of comprehensive scores demonstrated that a 22.5% ethanol dosage optimally balanced wetting efficiency and granule compactness, thereby establishing it as the optimal parameter.

**Table 5:** Effects of ethanol dosage on granulation performance

Ethanol dosage (%)	Molding ratio (%)	Angle of repose score	Bulk density (g/100 mL)	Dissolution ratio (%)	Comprehensive score
7.5	56.19	100	41.18	72.93	67.35
12.5	73.22	100	43.22	66.78	73.93
17.5	88.56	80	40.17	70.11	69.71
22.5	97.99	100	49.43	67.58	78.75
27.5	96.22	100	46.23	72.5	78.74

### 3.5 Optimization of drying temperature

To investigate the effects of drying temperature on granule properties, gradient experiments were conducted at 60 °C, 70 °C, and 80 °C (Table 6). The results demonstrated that the 70 °C group achieved the highest comprehensive score, significantly surpassing the 60 °C group (78.77) and 80 °C group (82.00). At 70 °C, the granules achieved the highest comprehensive score of 82.09, with a dissolution ratio of 80.23% and a bulk density of 49.81 g/100 mL. This indicates that moderate heating promotes uniform moisture evaporation and enhances granule solubility. However, the dissolution rate sharply declined to 75.88% at 80 °C, likely due to thermal degradation of heat-sensitive components or surface hardening of granules.^45^ Based on the temperature-score correlation, 70 °C was identified as the optimal drying temperature.

**Table 6:** Effects of drying temperature on granule properties

Drying temperature (°C)	Molding ratio (%)	Angle of repose score	Bulk density (g/100 mL)	Dissolution ratio (%)	Comprehensive score
60	97.99	100	49.43	67.66	78.77
70	98.31	100	49.81	80.23	82.09
80	99.06	100	49.06	75.86	80.00

### 3.6 Optimization of drying time

By establishing a drying time gradient of 60–90 minutes (Table 7), the 60 minutes group achieved the highest comprehensive score (82.18), with its dissolution rate (80.63%) and bulk density (49.8 g/100 mL) obviously outperforming the 75 minutes and 90 minutes groups. Extending drying time to 90 minutes resulted in a partial recovery of dissolution rate (76.73%), but bulk density continued to decline, suggesting prolonged drying may disrupt internal porosity equilibrium.^46,47^ The ranking of comprehensive scores indicated that the 60 minutes drying time optimally balanced critical parameters such as granulation yield, dissolution rate, and bulk density, thereby maximizing the retention of granule functional structure and stability.

**Table 7:** Effects of drying time on granule properties

Drying time (min)	Molding ratio (%)	Angle of repose score	Bulk density (g/100 mL)	Dissolution ratio (%)	Comprehensive score
60	98.3	100	49.8	80.63	82.18
75	99.05	100	48.59	67.48	78.78
90	98.59	100	45.78	76.73	80.28

### 3.7 Preserving dissolution, are consistent withts

As shown in Table 8, aspartame exhibits a sweetness intensity 200 times that of sucrose, with a pure sweet taste and refreshing aftertaste, free from metallic notes. Sucralose closely mimics the sweetness profile of sucrose, while mannitol, a low-calorie sugar alcohol, has a sweetness intensity of 0.7 times that of sucrose. Stevioside, though 350–450 times sweeter than sucrose, imparts a lingering bitter aftertaste.^48,49^

**Table 8:** Characteristics of sweeteners

Sweetener	Sweetness intensity (*vs.* Sucrose)	Properties
Aspartame	200×	Pure sweetness, refreshing aftertaste, no metallic notes, flavor-enhancing effect
Sucralose	600–650×	Sweetness profile and aftertaste closely resemble sucrose
Mannitol	0.7×	Low-calorie sugar alcohol, sucrose-like sucrose
Stevioside	350–450×	High sweetness intensity with lingering bitter aftertaste

Table 9 indicates that the addition of 0.5% aspartame and 0.2% sucralose achieved the highest sensory scores. Based on the principle of minimal dosage, 0.2% sucralose was selected as the optimal sweetener. And strawberry flavor at 0.8% yielded the highest average score (2.5 points), delivering a rich and pleasant flavor profile that significantly enhanced palatability and patient acceptability. Combining the sensory evaluation results of sweeteners and flavoring agents, the final formulation incorporated 0.2% sucralose and 0.8% strawberry flavor to optimize the taste and aroma of XLGs.

**Table 9:** Sensory evaluation results of sweeteners and flavoring agents

Additive category	Additive	Dosage (%)	Average score
Sweetener	Mannitol	0.5	0.2
		1.0	0.3
		2.0	0.5
	Stevioside	0.1	0.8
		0.3	1.0
		0.5	1.8
		0.7	0.5
	Aspartame	0.1	0.8
		0.3	1.7
		0.5	2.3
		0.7	0.6
	Sucralose	0.1	0.9
		0.2	2.7
		0.4	1.0
		0.6	0.8
Flavoring agent	Red apple flavor	0.1	0.4
		0.3	1.6
		0.5	1.1
	Strawberry flavor	0.1	0.8
		0.5	1.6
		0.8	2.5
		1.0	1
	Sweet orange flavor	0.1	0.7
		0.5	1.5
		1.0	0.9

It is important to clarify the scope of the acceptability evidence presented in this study. The sensory evaluation of sweeteners and flavoring agents was conducted with adult volunteers and provides formulation-level optimization data. The caregiver survey complements this by offering consumer-oriented evidence on real-use barriers and expected preferences. However, we acknowledge that neither approach constitutes definitive pediatric intake validation. Prospective palatability studies with repeated-dose administration in the target pediatric population remain necessary to fully establish actual child acceptance and compliance.

A total of 50 valid caregiver questionnaires were collected from families whose children had previously used Xiaoer Lingyin Decoction (Table 10). The traditional decoction was associated with clear administration barriers, 84.0% of caregivers reported unpleasant bitterness/astringency, 78.0% considered the administration volume inconvenient, 82.0% reported preparation inconvenience, and 76.0% considered storage or portability inconvenient. In addition, 70.0% of caregivers reported child refusal, delayed intake, or difficulty completing the dose, suggesting that the decoction form may negatively affect pediatric medication adherence.

**Table 10:** Caregiver-reported barriers to traditional Xiaoer Lingyin decoction and expected acceptability of taste-masked XLGs^a^

Category	Survey item	Agree/strongly agree, *n* (%)	Mean score
Administration barriers of traditional decoction	The decoction has an unpleasant bitter/astringent taste	42 (84.0%)	4.35 ± 0.68
	The administration volume is inconvenient for children	39 (78.0%)	4.10 ± 0.75
	Preparation of the decoction is time-consuming or inconvenient	41 (82.0%)	4.22 ± 0.72
	Storage or portability of the decoction is inconvenient	38 (76.0%)	4.04 ± 0.80
	The child refused, delayed, or had difficulty completing the dose	35 (70.0%)	3.90 ± 0.86
	Caregivers were concerned about poor adherence during repeated administration	37 (74.0%)	3.98 ± 0.84
Expected acceptability of granule conversion	Granules would be more convenient than the traditional decoction	45 (90.0%)	4.50 ± 0.60
	Small-volume warm-water administration would be more suitable for children	43 (86.0%)	4.38 ± 0.64
	Taste masking may improve children's willingness to take the medicine	44 (88.0%)	4.45 ± 0.62
	Caregivers would prefer taste-masked XLGs if available	43 (86.0%)	4.40 ± 0.66
	Taste-masked granules would be more suitable for outpatient or home use	42 (84.0%)	4.30 ± 0.70
Taste-design preference	Strawberry flavor would be acceptable for taste-masked XLGs	40 (80.0%)	4.15 ± 0.74
	Excessively strong sweetness should be avoided	38 (76.0%)	4.02 ± 0.80

a Data are presented as *n* (%) of caregivers selecting “agree” or “strongly agree” and as mean ± SD based on a five-point Likert scale, where 1 = strongly disagree and 5 = strongly agree. XLGs, Xiaoer Lingyin granules. These data reflect caregiver-reported expected acceptability and do not represent direct repeated-dose pediatric intake validation.

Caregivers showed strong preference for granule conversion and taste masking. Specifically, 90.0% agreed that granules would be more convenient than the traditional decoction, 86.0% considered small-volume warm-water administration more suitable for children, and 88.0% believed that taste masking would improve children's willingness to take the medicine. Furthermore, 86.0% indicated that they would prefer taste-masked XLGs if available. Strawberry flavor was considered acceptable or preferable by 80.0% of caregivers, supporting its use in the optimized formulation.

These results provide caregiver-reported evidence supporting the pediatric-use rationale and expected acceptability of taste-masked XLGs, although prospective pediatric intake studies remain necessary for definitive validation.

### 3.8 Optimization of formulation process *via* response surface methodology

Based on the results of single factor test, it can be seen that the ratio of diluent to main drug, the concentration of wetting agent and the dosage of wetting agent are considered to be the three factors that have the greatest influence on the particle properties. Response surface methodology was used to investigate the optimal conditions of three factors, namely, the ratio of drug-to-excipient (A), the concentration of wetting agent (B) and the dosage of wetting agent (C), with molding ratio, angle of repose, bulk density and dissolution ratio as the indexes. The test results are shown in Table 11.

**Table 11:** Results of response surface experiments

Dextrin : drug ratio (A)	Ethanol concentration (%) (B)	Ethanol dosage (%) (C)	Molding ratio (%)	Angle of repose score	Bulk density (g/100 mL)	Dissolution ratio (%)	Comprehensive score
0.5 : 1.0	65	27.5	93.16	80	48.6	83.78	76.38
0.5 : 1.0	55	22.5	97.67	80	35	80.88	73.39
0.5 : 1.0	65	17.5	92.69	80	49.21	66.18	72.02
0.5 : 1.0	75	22.5	84.96	100	41.1	72.43	75.12
1.0 : 1.0	65	22.5	95.88	100	46.28	86.32	82
1.0 : 1.0	75	17.5	96.36	80	42.21	65.65	70.3
1.0 : 1.0	65	22.5	99.69	100	44.6	88.06	83.09
1.0 : 1.0	55	27.5	90.46	100	47.4	70.58	77.86
1.0 : 1.0	75	27.5	96.83	80	44.8	64.5	71.53
1.0 : 1.0	65	22.5	90.51	100	48.27	82.18	80.24
1.0 : 1.0	65	22.5	95.33	100	47.94	83.78	81.76
1.0 : 1.0	65	22.5	97.03	100	47.94	53.78	74.26
1.0 : 1.0	55	17.5	91.55	100	48.03	54.03	73.65
1.5 : 1.0	55	22.5	94.27	80	43.02	44.63	64.57
1.5 : 1.0	75	22.5	89.64	100	42.57	59.63	72.96
1.5 : 1.0	65	27.5	98.96	80	46.38	60	71.34
1.5 : 1.0	65	17.5	96.64	80	47.67	58.05	67.09

To further support the powder performance evaluation, the physical properties of dry extract powders prepared by different drying methods were further interpreted in combination with previously reported literature data. As shown in related studies on herbal extract powders, freeze-drying generally leads to the formation of highly porous and low-density structures, which significantly improves flowability and compressibility by reducing interparticle cohesion and enhancing powder rearrangement capability during compaction.^29,30^

Consistent with these findings, the freeze-dried Xiaoer lingyin extract exhibited lower bulk-related resistance and improved structural looseness compared with thermally dried counterparts. This structural advantage can be attributed to ice-crystal sublimation during freeze-drying, which generates a porous matrix that facilitates interparticle contact rearrangement and liquid bridge formation during subsequent granulation.

These observations further confirm that freeze-dried intermediate powders provide a more favorable physical foundation for granule formation.

The quadratic polynomial model was fitted using Design-Expert 13.0 software, yielding the following regression equation:Comprehensive score = −176.55687 + 44.31500*A* + 2.73312*B* + 7.16500*C* −0.079500*AB* − 0.416000*AC* − 0.001750*BC* − 15.32000*A*^2^ − 0.035825*B*^2^ − 0.141800*C*^2^where *Y* represents the predicted comprehensive score, *A* represents the drug-to-excipient ratio, *B* represents the ethanol concentration (% v/v), and *C* represents the ethanol dosage (%).

The statistical significance and adequacy of the fitted quadratic regression model were evaluated by analysis of variance (ANOVA). As shown in Table 12, the overall model was highly significant, with an *F*-value of 32.87 and a *p*-value of less than 0.0001, indicating that the fitted model was statistically reliable. Among the linear terms, ethanol dosage (*C*) had a significant effect on the comprehensive score (*p* = 0.0037), whereas the drug-to-excipient ratio (*A*) and ethanol concentration (B) were not significant within the investigated range, with *p*-values of 0.1949 and 0.1915, respectively. For the interaction terms, *AC* was statistically significant (*p* = 0.0427), indicating that the combined variation of the drug-to-excipient ratio and ethanol dosage affected the comprehensive score. In contrast, *AB* and *BC* were not significant, with *p*-values of 0.3761 and 0.8411, respectively. The quadratic terms *A*^2^, *B*^2^, and *C*^2^ were all highly significant (*p* < 0.0001), suggesting that the response was strongly influenced by the curvature effects of the three formulation variables.

**Table 12:** Analysis of variance (ANOVA)

Source	Sum of squares	df	Mean square	*F*-value	*p*-value	Significance
Model	209.38	9	23.26	32.87	<0.0001	Significant
*A*-Excipient ratio	1.45	1	1.45	2.05	0.1949	Not significant
*B*-Ethanol concentration	1.48	1	1.48	2.09	0.1915	Not significant
*C*-Ethanol amount	12.93	1	12.93	18.27	0.0037	Significant
*AB*	0.6320	1	0.6320	0.8930	0.3761	Not significant
*AC*	4.33	1	4.33	6.11	0.0427	Significant
*BC*	0.0306	1	0.0306	0.0433	0.8411	Not significant
*A* ^2^	61.76	1	61.76	87.27	<0.0001	Significant
*B* ^2^	54.04	1	54.04	76.36	<0.0001	Significant
*C* ^2^	52.91	1	52.91	74.77	<0.0001	Significant
Residual	4.95	7	0.7077	—	—	—
Lack of fit	0.3615	3	0.1205	0.1049	0.9529	Not significant
Pure error	4.59	4	1.15	—	—	—
Cor total	214.34	16	—	—	—	—

The lack-of-fit test was not significant, with an *F*-value of 0.1049 and a *p*-value of 0.9529, indicating that the model did not exhibit significant lack of fit relative to the pure error. Therefore, the fitted quadratic model was considered adequate for describing the relationship between the formulation variables and the comprehensive score.

The goodness-of-fit statistics further confirmed the reliability of the model. As shown in Table 13, the *R*^2^ value was 0.9769, indicating that 97.69% of the variation in the comprehensive score could be explained by the fitted model. The adjusted *R*^2^ and predicted *R*^2^ values were 0.9472 and 0.9395, respectively, and the difference between them was less than 0.2, suggesting good agreement between the fitted and predicted responses. The C.V. value was 1.11%, indicating good experimental precision and low variability. In addition, the adequate precision value was 14.3517, which was higher than the recommended threshold of 4, confirming an adequate signal-to-noise ratio. These results demonstrated that the quadratic model was reliable for predicting the comprehensive score and navigating the formulation design space.

**Table 13:** Fit statistics of the fitted quadratic response surface model

Parameter	Std. Dev.	Mean	C.V. (%)	*R* ^2^	Adjusted *R*^2^	Predicted *R*^2^	Adequate precision
Value	0.8413	75.91	1.11	0.9769	0.9472	0.9395	14.3517

**Fig. 6:**
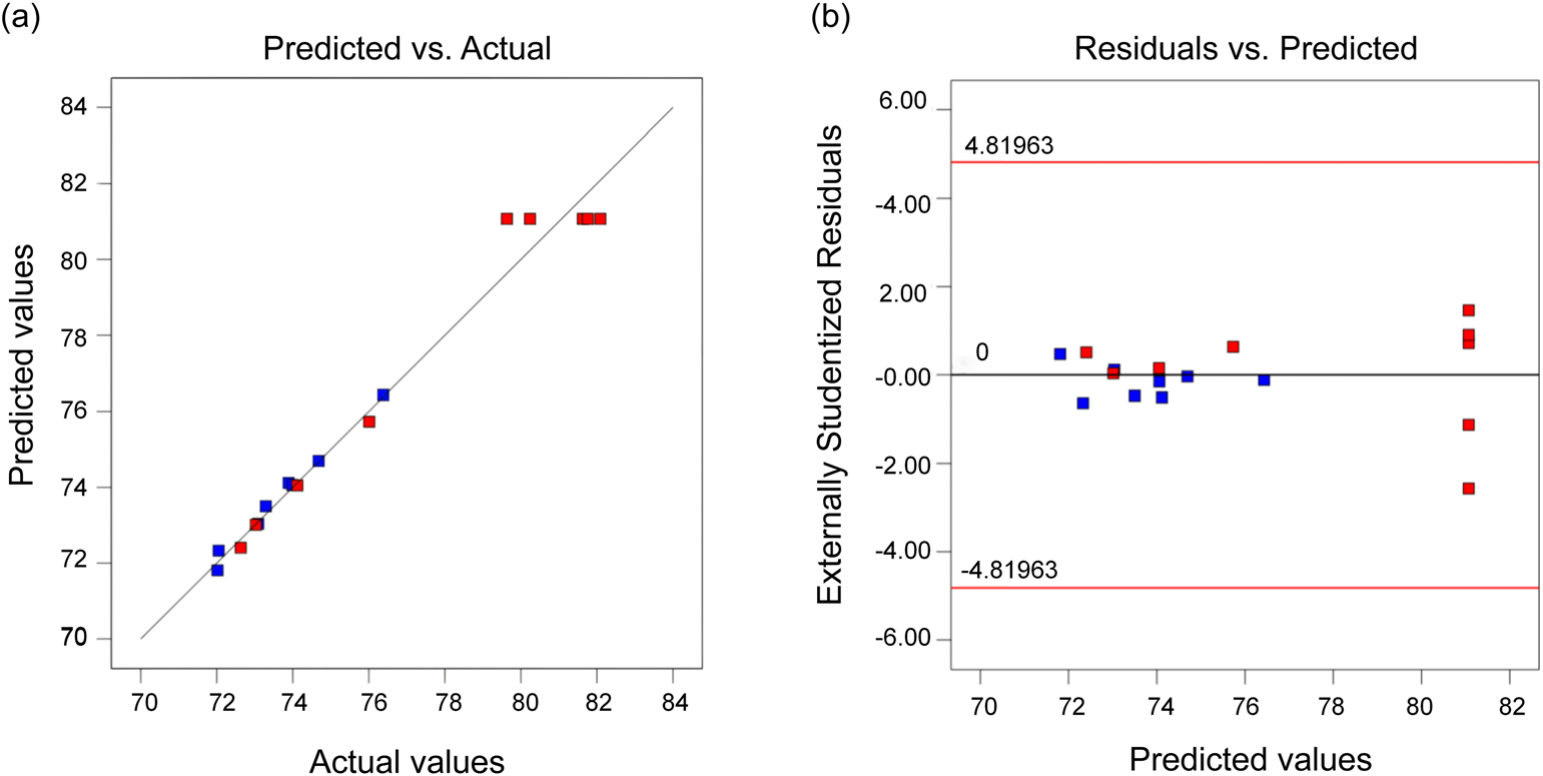
Diagnostic plots for validation of the fitted quadratic response surface model: (a) predicted *versus* actual values of the comprehensive score; (b) residuals *versus* predicted values. The agreement between predicted and actual values and the random distribution of residuals around zero indicate the adequacy and predictive reliability of the model.

To further validate the predictive performance of the model, diagnostic plots were generated. As shown in Fig. 6a, the predicted values were in good agreement with the actual values, with most data points distributed close to the diagonal reference line. The residuals *versus* predicted values plot in Fig. 6b showed that the residuals were randomly distributed around zero without an obvious systematic trend. These results further confirmed the adequacy and predictive reliability of the fitted response surface model.

**Fig. 7:**
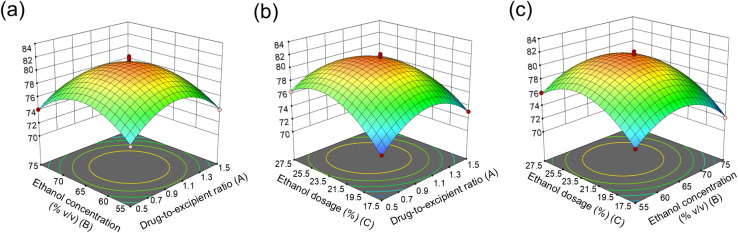
Response surface plots showing the interaction effects of formulation variables on the comprehensive score: (a) drug-to-excipient ratio (A) and ethanol concentration (B); (b) drug-to-excipient ratio (A) and ethanol dosage (C); (c) ethanol concentration (B) and ethanol dosage (C). Factor A represents the drug-to-excipient ratio, factor B represents ethanol concentration (% v/v), and factor C represents ethanol dosage (%).

Response surface plots were then used to visualize the interaction effects among the three formulation variables. As shown in Fig. 7, the AC interaction had the most pronounced and statistically significant effect on the comprehensive score (*p* = 0.0427), whereas the AB and BC interactions were not significant. This result indicates that the drug-to-excipient ratio and ethanol dosage jointly regulated granule quality. From a physicochemical standpoint, this interaction may be attributed to the balance between excipient-mediated particle dispersion and wetting-agent-induced liquid bridge formation. An appropriate drug-to-excipient ratio provides sufficient solid surface and interparticle contact points, while a suitable ethanol dosage promotes liquid bridge formation and granule consolidation. Insufficient wetting may result in poor cohesion and low molding ratio, whereas excessive wetting may cause overwetting, reduced porosity, and impaired dissolution. Therefore, only specific A–C combinations can simultaneously improve molding performance, flowability, and dissolution behavior.

Based on the fitted model, the optimal formulation conditions were predicted as follows: drug-to-excipient ratio = 0.961 : 1, ethanol concentration = 64.43%, and ethanol dosage = 23.46%, with a predicted comprehensive score of 81.22. Considering practical operability, the optimized formulation was adjusted to a drug-to-excipient ratio of 1 : 1, ethanol concentration of 65%, and ethanol dosage of 22.5%.

According to the above-mentioned optimal prescription, three batches of process verification were carried out, and the results showed that the scores of the three verification batches were 80.96–81.21 (Table 14), showing the smallest average relative error (0.17%), thus confirming that the model can accurately predict the prescription ratio of XLGs.^18^

**Table 14:** Validation results of the optimal formulation

Batch	Molding ratio (%)	Angle of repose score	Bulk density (g/100 mL)	Dissolution ratio (%)	Actual comprehensive score	Predictive comprehensive score	Average relative error (%)
1	97.87	100	46.01	79.98	80.96	81.22	0.17%
2	97.86	100	46.34	80.12	81.08		
3	98.45	100	46.63	79.76	81.21		

From a physicochemical standpoint, the dominant *A* × *C* interaction arises because the binder requirement is not fixed but scales with the specific surface area and porosity defined by the drug-to-excipient ratio (A). A higher drug fraction typically increases surface area and interparticle friction, necessitating more wetting agent (C) to form sufficient liquid bridges and achieve granule consolidation; conversely, a lower drug fraction (*i.e.*, more excipient) reduces the binder demand, and excessive *C* can push the system toward overwetting, coalescence, and loss of flow. Thus, A governs the number and topology of potential contact points and capillaries, whereas *C* dictates the liquid saturation and bridge strength; their coupling determines the transition between pendular/funicular states, which directly controls nucleation, growth, and breakage during granulation. This mechanism explains why the *A* × *C* term most strongly impacts our critical quality attributes, including molding ratio (*via* consolidation efficiency), angle of repose (*via* interparticle friction and size distribution), and dissolution performance (*via* porosity and pore connectivity), and why only specific *A*–*C* combinations yield simultaneously robust flowability and rapid dissolution.^50,51^

### 3.9 Preliminary stability evaluation of optimized granules

**Fig. 8:**
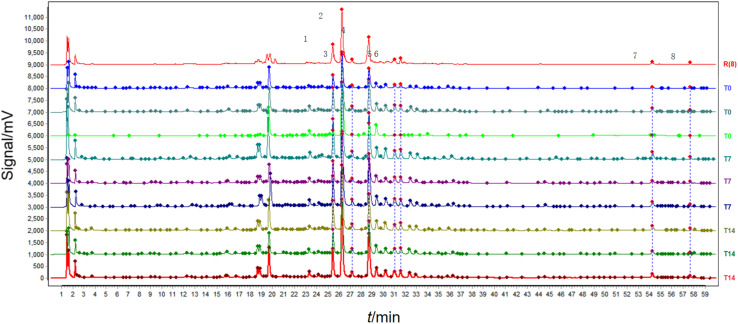
UPLC fingerprint chromatograms of optimized freeze-dried XLGs at T0 (0 days), T7 (7 days), T14 (14 days). The chromatographic profiles showed high consistency, with similarity values of 0.999 (T7 *vs.* T0) and 0.998 (T14 *vs.* T0).

The chemical stability of the optimized XLGs was evaluated by monitoring UPLC fingerprint changes over 14 days. UPLC fingerprint analysis was performed at each time point. As shown in Fig. 8, the chromatographic profiles remained highly consistent across the three time points, with no new peaks observed and no obvious changes in the major peak patterns. Using T0 as the reference chromatogram, the similarity values were 0.999 and 0.998 for T7 and T14, respectively, indicating acceptable chemical stability during the 14 days storage period.

Quantitative analysis of the four representative marker compounds showed high retention rates over the 14 days period. The retention values of all four markers remained within 98.5–101.2% of their initial levels, indicating no marked degradation of the representative constituents during short-term storage (Table 15).

**Table 15:** Retention of marker compounds in optimized freeze-dried XLGs during short-term storage (*x̄* ± *s*, *n* = 3)^a^

Marker compound	Initial content at T0 (mg g^−1^)	T7 Retention (% of T0)	T14 Retention (% of T0)
Neoastilbin	4.158 ± 0.040	99.2 ± 1.1	98.7 ± 1.3
Astilbin	11.806 ± 0.052	100.1 ± 0.8	99.5 ± 1.0
Irigenin	1.630 ± 0.037	99.8 ± 1.2	98.5 ± 1.5
Irisflorentin	0.241 ± 0.046	100.5 ± 1.4	101.2 ± 1.8

a Retention values were calculated relative to the corresponding T0 value of each batch. Slight values above 100% were considered to be within analytical variability.

Moisture content was monitored throughout the storage period. The initial moisture content of the optimized freeze-dried granules was 5.8 ± 0.3% (w/w), which increased slightly to 6.1 ± 0.4% at T7 and 6.3 ± 0.5% at T14. No pronounced moisture uptake was observed during storage, indicating acceptable physical stability under the tested conditions.

These preliminary results indicate that the optimized freeze-dried XLGs maintain acceptable chemical and physical stability under ambient conditions for at least 14 days. Long-term stability studies under ICH conditions (25 °C/60% RH and 40 °C/75% RH) will be conducted in future work to fully establish the shelf-life of the formulation.

## 4 Conclusion

This study developed and optimized Xiaoer Lingyin granules using single-factor experiments combined with RSM. The optimized process consisted of a drug-to-excipient ratio of 1 : 1, 65% (v/v) ethanol, 22.5% ethanol dosage, drying at 70 °C for 60 min, 0.2% sucralose, and 0.8% strawberry flavoring. The optimized granules showed acceptable molding performance, flowability, dissolution behavior, and palatability. The fitted RSM model was statistically reliable, as supported by ANOVA, lack-of-fit analysis, *R*^2^-related statistics, diagnostic plots, and validation experiments.

The main contribution of this study is the establishment of a multi-index formulation optimization strategy for pediatric TCM granules. Unlike optimization based on a single quality attribute, this approach integrates granule formation, powder flowability, dissolution performance, and sensory acceptability, thereby linking manufacturing quality with pediatric medication compliance. These findings provide a practical and reproducible basis for the modernization and standardization of XLGs. It should be noted that the present study did not include direct SEM observation, particle-size distribution analysis, or detailed surface characterization of the optimized granules. These microstructural analyses will be addressed in future work to further elucidate the structure–property relationships of XLGs. Future studies should further evaluate long-term stability, bioactive component retention, pharmacokinetics, and scale-up feasibility. In addition, this study did not conduct systematic toxicological evaluation or pediatric clinical trials. While the formulation is based on a clinically used traditional Chinese medicine prescription and employs pharmaceutically acceptable excipients, further safety assessment and clinical validation are required to support its translation into pediatric clinical practice. Furthermore, while caregiver-reported acceptability data support the expected pediatric suitability of taste-masked XLGs, direct repeated-dose palatability studies in children were not performed and are planned for future work.

## Ethical statement

The survey was reviewed and approved by the Ethics Committee of Guizhou University of Traditional Chinese Medicine (Approval No. 2024064).

## Consent to participate

Informed consent was obtained from all individual participants or their legal guardians prior to their enrollment.

## Author contributions

Yanru Liu: conceptualization, formal analysis, investigation, methodology, validation, visualization, writing – original draft, writing – review and editing. Taoyang Cai: conceptualization, investigation, resources, methodology, validation, visualization, writing – original draft, writing – review and editing. S.K. Kot-Cheung: conceptualization, supervision, writing – original draft, writing – review and editing. Xinli Song: project administration, supervision, writing – original draft, writing – review and editing. Feixiong Chen: project administration, supervision, writing – original draft, writing – review and editing. All authors have read and agreed to the published version of the manuscript.

## Conflicts of interest

The authors declare no conflicts of interest.

## Data Availability

Data are available on request to the authors.
